# Comparative Orbital Volumes between a Single Incisional Approach and a Double Incisional Approach in Patients with Combined Blowout Fracture

**DOI:** 10.1155/2015/982856

**Published:** 2015-04-16

**Authors:** Hyun Ho Han, Sang Wook Park, Suk-Ho Moon, Bommie F. Seo, Jong Won Rhie, Sang Tae Ahn, Deuk Young Oh

**Affiliations:** Department of Plastic Surgery, College of Medicine, The Catholic University of Korea, Seoul 137-701, Republic of Korea

## Abstract

*Purpose*. Blowout fracture characterized by concurrent floor and medial wall fractures is a rare entity. We compared surgical outcomes between a single approach and a double approach in patients with orbital fracture by measuring the postoperative orbital volume. *Methods*. We confirmed that 21 (8.5%) of a total of 246 patients with orbital fractures had fractures of the medial wall and floor through a retrospective chart review. Of these, 10 patients underwent the single approach and the remaining 11 patients had the double approach. We performed a statistical analysis of changes between the preoperative and postoperative orbital volumes at a 6-month follow-up. *Results*. Compared with the contralateral, nonaffected side, the orbital volume was 115.3 (±6.09)% preoperatively and 106.5 (±6.15)% postoperatively in the single approach group and 118.2 (±11.16)% preoperatively and 108.6 (±13.96)% postoperatively in the double approach. These results indicated that there was a significant difference between the preoperative and postoperative orbital volumes in each group (*P* < 0.05). However there was no significant difference between the single approach and the double approach (*P* > 0.05). *Conclusions*. Our results showed that there were no significant differences in surgical outcomes between the two modalities. The treatment modality may be selected based on the surgeons' preference, as well as the fracture type.

## 1. Purpose

Blowout fractures most commonly occur in the medial wall or floor of the orbital wall. Repair should be performed in cases of extraocular muscle entrapment, diplopia, globe malposition, and significant orbital volume expansion [1–5]. Cases of blowout fracture involving both the medial wall and the floor are rare and the suitable surgical approaches are debatable [6].

The surgical approach according to the fracture sites of the floor is* via* subciliary, transconjunctival, or subtarsal incision [7]. Additionally, the approach for the medial wall fractures is* via* the transcaruncular [8, 9], Lynch incision [10], or endonasal approach [11, 12]. Alternatively, by the extension of the subciliary or transconjunctival incision of the floor or transcaruncular incision to the medial side, a single incision can be made to achieve reduction of both floor and medial wall fractures [13].

A double approach is useful to secure the visualization of the entire fracture in each site of fracture. There is a high possibility that the insertion site for the inferior oblique muscle might be preserved. This leads to the remote possibility that the postoperative diplopia might occur but may prolong the operation time. The single approach has the advantage of a single incision for the reduction of 2 fracture sites. This approach shortens the operation time but may not be useful to secure a visual field. This may lead to incomplete reduction and the inaccurate placement of the implant.

We compared surgical outcomes between a single approach and a double approach in patients with orbital fracture and measured the postoperative orbital volume. We performed a comparative analysis of the degree of the recovery of the orbital volume.

## 2. Method

**Figure 1:**
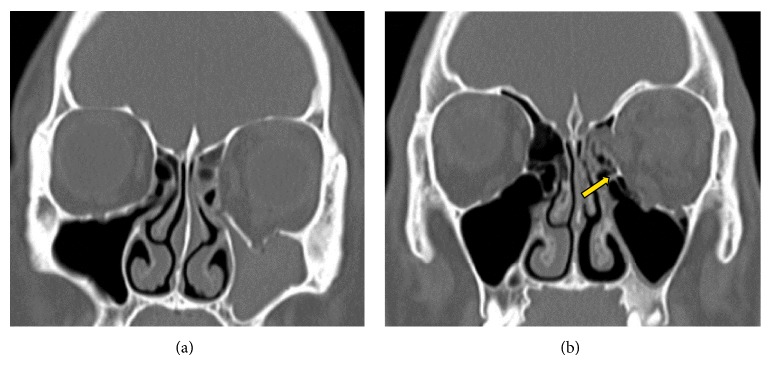
(a) We excluded patients with inferomedial fracture where there was a connection between medial and floor fractures due to the concurrent fractures of inferomedial strut, the ethmoid-maxillary junction. (b) Fractures in floor and medial wall without the inferomedial strut (yellow arrow) only have been included in this study.

Seoul St. Mary's Hospital Institutional Review Board approved the current study. A retrospective chart review during the period from January, 2009, to December, 2013, confirmed that 21 (8.5%) of a total of 246 patients with orbital fractures had fractures of the medial wall and floor. All patients sustained an injury due to trauma, and they presented with no notable findings in the eye balls. Patients who underwent surgery within 2 weeks of the onset of injury were enrolled in the current study. Furthermore, we excluded patients with inferomedial fracture where there was a connection between the medial and floor fractures due to the concurrent fractures of inferomedial strut, the ethmoid-maxillary junction (Figure 1). Of the 21 patients, 11 underwent a double approach* via* a transcaruncular and a subciliary incision and the remaining 10 had a single approach* via* a subciliary incision. The single incisional approach was used during the initial 3 years from 2009 to 2011. From 2012, double incisional approach was used to obtain more wide surgical field. All the surgical procedures were performed by a single surgeon (D.Y.O.). Porous polyethylene (PPE) was used in the placement of the implant.

We evaluated the operation time. We also analyzed the postoperative complications such as visual acuity, diplopia, extraocular muscle limitation, vertical globe position, and hertel exophthalmometry. We performed a CT scan 6 months postoperatively. We completed the treatment after a 12-month follow-up in patients who did not show an eventful postoperative course.

### 2.1. Surgical Technique

**Figure 2:**
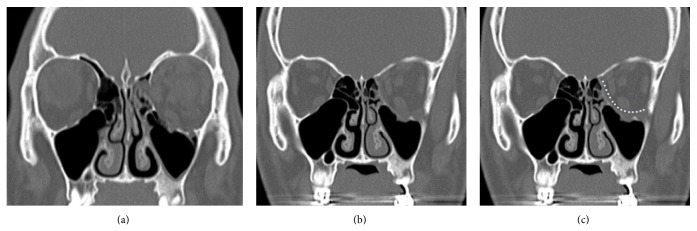
Single incisional approach. (a) A 27-year-old male patient was injured by a baseball on his left orbit wall. (b) A single approach was done* via* a subciliary incision. (c) A single layer of the porous polyethylene was used to cover the defects in the floor and on the medial wall, similar to the wrap-around technique described by Nunery et al.

All patients underwent surgical procedure under general anesthesia. The patients underwent forced duction test preoperatively as an evaluation of globe restriction. The floor was opened* via* a subciliary incision in the single approach. A periosteum incision line was extended to the inferomedial strut, if at least it was possible to obtain a closer visual identification of the medial wall. Once the operative field was sufficiently secured, sufficient reduction of the soft tissue at sites of the floor fracture was achieved. For better visualization of medial wall, the patients were tilted head down and turned his/her head to the opposite direction from injured orbit. An exposure to frontoethmoidal suture is also possible. We confirmed the medial wall fracture and achieved a sufficient reduction of the soft tissue. We subsequently inserted porous polyethylene (PPE) of 1 mm thickness. Similar to the wrap-around technique described by Nunery et al. [14], a single layer of PPE was used to cover the defects in the floor and on the medial wall. Moreover, we ensured that the implants were sufficiently placed in the anterior, lateral, and posterior margins of the fracture (Figure 2).

**Figure 3:**
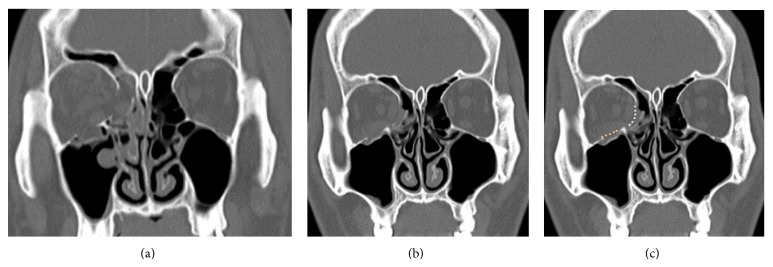
Double incisional approach. (a) A 36-year-old male patient was injured by a fist injury. (b) A double approach was done* via* subciliary incision and transcaruncular incision. (c) Each implant (porous polyethylene) was placed in the fracture sites on the medial wall and floor.

A subciliary incision was made for the reduction of the floor, as described above, followed by a transcaruncular incision, in the double approach. With the minimal dissection of the inferomedial strut, efforts were made not to detach the insertion sites of the inferior oblique muscle. In contrast, in case of double-approach, two separate implants were used for reconstruction. Each implant was placed in the fracture sites on the medial wall and floor. Moreover, we ensured that the implants were sufficiently placed in the anterior, lateral, and posterior margins of the fracture (Figure 3). We also examined the proportion of the postoperative diplopia, as compared with a single approach.

We performed a forced duction test postoperatively, after the implantation, to determine whether there were restrictions to the globe mobility.

### 2.2. The Measurement of the Orbital Volume

We measured the orbital volume, as previously described in the literature [15–18]. Changes between the preoperative and postoperative orbital volumes were analyzed by CT scan imaging. CT scans of all patients were taken 6 month postoperatively. Even though PPE is a nonradiopaque material, it is visualized easily through adjusting CT density. The orbital volume at surgical sites was measured using Centricity Radiology RA 600 Clinical v8.0 (GE Medical System, WI, USA). The volume was obtained by measuring the surface of the orbital margin drawn on a 3 mm thickness coronal section image. The origin of the orbital margin began from a point where the entire margin of the orbital cavity could be seen on cross section to the point where the margins converged. A single observer who measured the orbital volume blinded to the technique used measured the orbital volume twice and then averaged the results to reduce measurement errors. We calculated the mean of both preoperative and postoperative values of the orbital volume assuming that there was no significant difference in the preoperative and postoperative orbital volume on the contralateral, nonaffected side. This served as the control group. Moreover, we also confirmed the degree of the difference in the orbital volume as compared with the affected side. We analyzed the decrease between preoperative and postoperative orbital volumes by a single approach and a double approach with the one sample *t*-test. Additionally, we also used the independent *t*-test to compare the postoperative orbital volume between the 2 groups. Statistical analysis was performed by the SPSS version 13.0 software (SPSS Inc., Chicago, IL, USA).

## 3. Result

21 (8.5%) of a total of 246 patients with orbital fractures had fractures of the medial wall and floor. 40 (16.3%) patients with fractures that included inferomedial strut were excluded from this study. A single approach was performed in 9 men and 1 woman. A double approach was performed in 9 men and 2 women. Average follow-up duration was 1 year 2 months.

Postoperative outcomes were summarized in Table 1. The mean operation time was 1 hour and 9 minutes in the single approach group and 1 hour and 48 minutes in the double approach group. The postoperative visual acuity was normal in all surgical patients. There was no postoperative extraocular muscle limitation. The vertical globe position was normal as compared with the contralateral side. Five patients (50%) from the single approach group had preoperative diplopia. Three patients (30%) had persistent diplopia for a month postoperatively. Seven patients (64%) from the double approach group had diplopia, of which 1 patient (9%) had persistent diplopia during the 1-month postoperative period. The diplopia disappeared after 1 month postoperatively in both groups. One patient each, in the single approach group (10%) and the double approach group (9%), presented with findings that were suggestive of enophthalmos characterized by a more than 2-mm difference on exophthalmometry. There were no other major complications during the follow-up period.

Compared with the contralateral, nonaffected side, the orbital volume was 115.3 (±6.09)% preoperatively and 106.5 (±6.15)% postoperatively in the single approach group and 118.2 (±11.16)% preoperatively and 108.6 (±13.96)% postoperatively in the double approach. These results indicated that there was a significant difference between the preoperative and postoperative orbital volumes in each group (*P* < 0.05). However, there was no significant difference between the single approach and the double approach (*P* > 0.05) (Tables 2 and 3).

## 4. Discussion

Several methods have been introduced for the treatment of blowout fracture, to date. The surgical technique additionally varies depending on the preference of surgeons. The pros and cons of each surgical modality are still controversial. Blowout fracture characterized by concurrent floor and medial wall fracture is a rare entity. Many controversial opinions exist regarding the surgical approaches and methods. We performed surgical operations by combining the single approach with the double approach method.

Both surgical approaches have their own merits and demerits. A single approach is routinely made by making a subciliary or transconjunctival incision, followed by the dissection. The length of the periosteum incision was extended to the inferomedial strut. We thus secured the surgical view up to the sites of the medial wall fracture. A single approach is advantageous since it may shorten the operation time by making a single incision for 2 fracture sites. However, it is disadvantageous in securing the surgical view as compared with a double approach. It is possible to resolve this by widening the surgical field* via* disinsertion of the inferior oblique muscle. There is, however, an increased risk of developing postoperative diplopia [19].

A double approach is advantageous in securing the visualization of the entire fracture, reducing the fracture sites, and inserting the implant. There is a remote possibility that it might cause the disinsertion of the inferior oblique muscle. This leads to a reduced risk of the postoperative diplopia, but the prolonged operation time remains problematic.

A single approach remains disadvantageous since it is difficult to secure the visual field, which requires detachment of the inferior oblique muscle from the bone insertion. Postoperative diplopia occurs in these cases. However, if the detached inferior oblique muscle is repaired by suturing, the incidence of the postoperative diplopia would be reduced [20]. Cho and Davies [19] reported that there is a possibility that mild vertical or torsional diplopia might transiently occur without performing the repair suture for the detached inferior oblique muscle, but these complications disappear within 1-2 weeks. As shown in the current results, the incidence of postoperative diplopia was higher in the single approach group as compared with the double approach group (30%* versus* 9%). We experienced no patients with a residual presence of the diplopia after 1 month postoperatively. Postoperative transient diplopia would not be problematic if the patients were preoperatively informed of the possibility of the complication. Additionally, the patients' head was lowered and then tilted to the medial side. This was sufficient to expose the upper and posterior portion of the medial orbital wall that was not observed in a single approach. Thus, problems with operative field were resolved to some extent.

It would not be acceptable to decide successful surgical outcome based on the orbital volume, as shown in the study design. Other authors have frequently used the same methods in conducting their studies in this series [15–18]. We collected the data about outcomes of surgical operations that had been performed by a single surgeon, to exclude other factors that may affect the results of the current study. Moreover, we solely analyzed cases with trauma etiologies. Furthermore, we enrolled only patients with a similar duration from the onset of trauma to the date of surgery. We notably excluded cases of inferomedial wall fracture, characterized by the fracture of the inferomedial strut, from the current analysis. Fractures extending from the floor to the medial wall require extensive coverage using implants. This poses challenging problems to surgeons [14]. The technical difficulty of the surgical operation may be a contributing factor to postoperative outcomes.

We demonstrated that both methods were effective in reducing the increased orbital volume due to the fracture. Our results also showed that there were no significant differences in surgical outcomes, except for the temporary occurrence of diplopia between the 2 modalities.

We concluded from our results that both surgical modalities were effective for the treatment of patients with fractures of the orbit wall and medial wall and furthermore the surgical treatment can be selected based on the surgeons' preference, as well as the fracture type.

## Figures and Tables

**Table 1:** Postoperative outcome.

	Single approach	Double approach
Operation time	1 hour 9 minutes	1 hour 48 minutes
Visual acuity	All were normal	All were normal
Extraocular muscle limitation	None	None
Vertical globe position	All were normal	All were normal
Temporary diplopia	Before operation: 5/10 (50%) *→*after operation: 10 (30%)	Before operation: 7/11 (64%) *→*after operation: 1/11 (9%)
Permanent diplopia	None	None
Enophthalmos	1/10 (10%)	1/11 (9%)

**Table 2:** Orbital volume, single approach.

Patient number	Preoperative volume increase (%) $\left(\frac{\text{preoperative injured orbit volume}}{\text{noninjured orbit volume}}\times 100\right)$	Postoperative volume increase (%) $\left(\frac{\text{postoperative injured orbit volume}}{\text{noninjured orbit volume}}\times 100\right)$	Difference (pre. − post.)
1	107	96	11
2	115	102	13
3	117	108	9
4	108	106	2
5	118	115	3
6	119	111	8
7	113	99	14
8	128	114	14
9	117	106	11
10	111	108	3
Average	**115.3**	**106.5**	**8.8**

**Table 3:** Orbital volume, double approach.

Patient number	Preoperative volume increase (%) $\left(\frac{\text{preoperative injured orbit volume}}{\text{noninjured orbit volume}}\times 100\right)$	Postoperative volume increase (%) $\left(\frac{\text{postoperative injured orbit volume}}{\text{noninjured orbit volume}}\times 100\right)$	Difference (pre. − post.)
1	126	119	7
2	135	125	10
3	110	102	8
4	122	109	13
5	132	132	0
6	116	101	15
7	125	122	3
8	98	87	11
9	114	102	12
10	116	100	16
11	106	96	10
Average	**118.2**	**108.6**	**9.5**

## References

[1] Dortzbach R. K. (1985). Orbital floor fractures. *Ophthalmic Plastic and Reconstructive Surgery*.

[2] Segrest D. R., Dortzbach R. K. (1989). Medial orbital wall fractures: complications and management. *Ophthalmic Plastic and Reconstructive Surgery*.

[3] Dutton J. J. (1991). Management of blow-out fractures of the orbital floor. *Survey of Ophthalmology*.

[4] Burnstine M. A. (2003). Clinical recommendations for repair of orbital facial fractures. *Current Opinion in Ophthalmology*.

[5] Harris G. J. (2006). Orbital blow-out fractures: surgical timing and technique. *Eye*.

[6] Su G. W., Harris G. J. (2006). Combined inferior and medial surgical approaches and overlapping thin implants for orbital floor and medial wall fractures. *Ophthalmic Plastic and Reconstructive Surgery*.

[7] Kothari N. A., Avashia Y. J., Lemelman B. T., Mir H. S., Thaller S. R. (2012). Incisions for orbital floor exploration. *The Journal of Craniofacial Surgery*.

[8] Morris D. E., Liliav B., Cohen M. N. (2014). Transcaruncular approach to the isolated medial orbital wall fracture: technical perspective and cadaveric dissection. *Journal of Craniofacial Surgery*.

[9] Edgin W. A., Morgan-Marshall A., Fitzsimmons T. D. (2007). Trancaruncular approach to medial orbital wall fractures. *Journal of Oral and Maxillofacial Surgery*.

[10] Garg S., Tao J., Burgett R., Nunery W. (2006). Supramid ‘sled’ implant repair of combined medial wall and orbital floor fracture. *Investigative Ophthalmology & Visual Science*.

[11] Chen C.-T., Chen Y.-R., Tung T.-C., Lai J.-P., Rohrich R. J. (1999). Endoscopically assisted reconstruction of orbital medial wall fractures. *Plastic and Reconstructive Surgery*.

[12] Sanno T., Tahara S., Nomura T., Hashikawa K. (2003). Endoscopic endonasal reduction for blowout fracture of the medial orbital wall. *Plastic and Reconstructive Surgery*.

[13] Scolozzi P. (2011). Reconstruction of severe medial orbital wall fractures using titanium mesh plates placed using transcaruncular-transconjunctival approach: a successful combination of 2 techniques. *Journal of Oral and Maxillofacial Surgery*.

[14] Nunery W. R., Tao J. P., Johl S. (2008). Nylon foil ‘wraparound’ repair of combined orbital floor and medial wall fractures. *Ophthalmic Plastic and Reconstructive Surgery*.

[15] Jung H. J., Kang S. J., Kim J. W. (2011). Quantitative analysis of the orbital volume change in isolated zygoma fracture. *Journal of the Korean Society of Plastic and Reconstructive Surgeons*.

[16] Ploder O., Klug C., Voracek M., Burggasser G., Czerny C. (2002). Evaluation of computer-based area and volume measurement from coronal computed tomography scans in isolated blowout fractures of the orbital floor. *Journal of Oral and Maxillofacial Surgery*.

[17] Ye J., Koung H. K., Sang Y. L. (2006). Evaluation of computer-based volume measurement and porous polyethylene channel implants in reconstruction of large orbital wall fractures. *Investigative Ophthalmology and Visual Science*.

[18] Kwon J., Barrera J. E., Jung T. Y., Most S. P. (2009). Measurements of orbital volume change using computed tomography in isolated orbital blowout fractures. *Archives of Facial Plastic Surgery*.

[19] Cho R. I., Davies B. W. (2013). Combined orbital floor and medial wall fractures involving the inferomedial strut: repair technique and case series using preshaped porous polyethylene/titanium implants. *Craniomaxillofacial Trauma and Reconstruction*.

[20] Rodriguez J., Galan R., Forteza G. (2009). Extended transcaruncular approach using detachment and repositioning of the inferior oblique muscle for the traumatic repair of the medial orbital wall. *Craniomaxillofacial Trauma and Reconstruction*.

